# Unpacking Health Literacy Disparities in Migrant and Ethnic Minority Communities in England

**DOI:** 10.1007/s10903-026-01861-8

**Published:** 2026-02-06

**Authors:** Carolina Machuca Vargas, Samia Turkistani, Anita David

**Affiliations:** School of Dental, Health and Care Professions, University of Portsmouth, Portsmouth, UK

**Keywords:** Health literacy disparities, Migrants communities, Health literacy questionnaire, Ophelia process

## Abstract

Health literacy is essential for navigating healthcare systems and making informed decisions. Migrant and minority ethnic populations often face barriers that impact their health literacy and access to care. This study explored the health literacy profiles and lived experiences of African and Latin American communities in England. A mixed-methods study was conducted using Phase 1 of the Ophelia (Optimising Health Literacy and Access) process. Sixty-five participants completed the Health Literacy Questionnaire (HLQ), and hierarchical cluster analysis identified distinct health literacy profiles. Two focus groups (n = 7 each) were held to explore participants’ experiences in greater depth. Qualitative data were analysed using thematic analysis. While participants demonstrated strengths in accessing and understanding health information, significant disparities emerged in areas requiring institutional engagement, trust in healthcare providers, and emotional support. Key barriers included digital exclusion, limited interpreter availability, fear of immigration consequences, and experiences of being dismissed or misunderstood in clinical settings. These challenges disproportionately affected asylum seekers and those with insecure immigration status, reflecting structural vulnerability and institutional racism. The study highlights the limitations of individual-level interventions and calls for system-wide changes to address the relational and structural barriers that undermine health equity. Improving health literacy among migrant populations requires more than accessible information. Equity-focused, co-designed interventions that confront institutional racism, invest in culturally safe communication, and prioritise emotional safety are essential to reducing racial and ethnic health disparities.

**Supplementary Information:**

The online version contains supplementary material available at https://doi.org/10.1007/s10903-026-01861-8.

## Background

Health literacy (HL) is the ability to obtain, comprehend, appraise, and apply health information to make informed decisions about health and care and has been increasingly recognised as a critical determinant of individual and population health outcomes [1, 2]. HL plays a fundamental role in enabling people to self-manage health conditions, engage meaningfully with healthcare providers, and navigate complex healthcare systems. Limited HL is consistently linked to poorer health status, lower engagement with preventive behaviours, increased hospitalisations, and higher mortality [3].

These challenges are particularly pronounced among migrants and minority ethnic populations, where HL intersects with structural inequalities, language barriers, cultural differences, and unfamiliarity with host country healthcare systems [4, 5]. Previous research has shown that migrants often face significant difficulties accessing care due to limited language proficiency, lack of culturally appropriate information, and complex service pathways [6, 7]. Language barriers, in particular, are known to negatively impact patient-provider communication and the quality of care received [8].

In the UK, despite policies aimed at promoting equitable care, migrants and minority ethnic groups continue to experience disparities in health outcomes and service access [9]. Structural barriers, such as complicated registration processes, inconsistent access to interpreters, and institutional biases, exacerbate these inequalities [10]. These challenges are further intensified by the increasing digitalisation of healthcare, which can marginalise individuals with low digital literacy or restricted internet access [11].

Refugees and asylum seekers face additional vulnerabilities due to trauma, financial instability, and uncertain legal status, which may limit their capacity to prioritise health and engage with healthcare services [12]. Emotional distress, fear of authority, and stigma around mental health further discourage help-seeking [13]. Interventions aimed at improving HL among these groups have highlighted the value of culturally tailored education, community engagement, and multilingual support [14, 15]. Strategies such as community health champions, peer-led training, and co-designed materials have shown promise in building trust and improving health outcomes [16, 17].

Portsmouth, a coastal city in the South East of England, exemplifies these national challenges at the local level. Nearly 15% of its residents identify as being from minority ethnic backgrounds [18], and the city hosts the highest number of asylum seekers and refugees in the region. Nonetheless, little is known about how these communities understand, engage with, and access healthcare. Understanding the health literacy strengths and needs of migrant and minority ethnic communities is essential for developing equitable, culturally responsive interventions and ensuring that services are accessible and capable of reducing health disparities. This study seeks to address this gap by exploring the HL levels, challenges, and support needs of migrants and minority ethnic communities in Portsmouth.

## Methods

### Study Design

This mixed-methods study followed Phase 1 of the Ophelia (Optimising Health Literacy and Access) process, a structured, evidence-based approach designed to improve health equity by identifying HL strengths and needs in underserved populations [19]. The Ophelia process is a systematic, equity-focused approach comprising three phases: identification of needs through health literacy profiling and qualitative enquiry, co-design of interventions with stakeholders, implementation and evaluation of those interventions [20].

This study presents findings from Phase 1 only, which included administration of the Health Literacy Questionnaire (HLQ), cluster analysis, and focus groups with members of low-health-literacy clusters to explore barriers and needs in greater depth.

## Setting and Participants

Participants were eligible if they were aged 18 years or older, identified as a migrant, asylum seeker, or refugee, and were able to provide informed consent. Recruitment was supported by two local organisations which have established trust within the community and experience working with underserved groups. All participants received an information sheet and provided written informed consent. Individuals who had difficulty reading English were offered the option of having the information read aloud by bilingual staff or community workers. Those who completed the survey and/or participated in a focus group were offered a shopping voucher as a token of appreciation.

The study received ethical approval from the University of Portsmouth Science and Health Faculty Ethics Committee (SHFEC 2022-085).

## Health Literacy Questionnaire (HLQ)

Health literacy was assessed using the Health Literacy Questionnaire (HLQ), a widely validated tool developed using grounded, validity-driven methodology [21]. The instrument contains 44 items across nine independent domains, reflecting a broad range of personal and system-level HL factors. The first five scales (e.g., *Feeling understood and supported by healthcare providers*) use a 4-point agreement scale, while the remaining four (e.g., *Ability to find good health information*) use a 5-point difficulty scale. The HLQ has been validated in diverse populations and is suitable for those with limited English proficiency and low literacy. In this study, the HLQ was administered in English or in an available translated version. Where an official translation did not exist for a participant’s preferred language, the questionnaire was read aloud by trained researchers in collaboration with community members to ensure cultural and linguistic accuracy. The questionnaire was administered in electronic and paper-based formats. In addition to HLQ items, the survey included basic demographic information (e.g., age, gender, ethnicity).

## Focus Groups

Participants from the two selected clusters (Clusters 3 and 4) were invited to join focus group discussions aimed at gaining deeper insights into their lived experiences of navigating health and care systems. They were purposively chosen because they represented the two most vulnerable health literacy profiles, with the lowest HLQ scores across multiple domains. Two focus groups were conducted in person and stratified by cultural background (one with primarily African participants, and the other with Latin American participants).

A vignette-based discussion guide was used to stimulate reflection and dialogue. A total of 5 vignettes (Supplementary file 1) were developed from the cluster profiles and described fictional but realistic scenarios that illustrated typical HL challenges (Table 1). This technique is consistent with the Ophelia methodology and facilitates accessible and culturally sensitive discussions [21].

Focus groups were moderated by trained researchers using a discussion guide to explore participants’ reactions to the vignettes and supported by community workers and interpreters when necessary (Supplementary file 1). Sessions were audio-recorded with consent, transcribed verbatim, and anonymised for analysis.

**Table 1:** Example of vignettes

“Gloria, a 40-year-old immigrant mum, works full-time to support her three children, aged 5 to 15, while coping with a recent separation. Her eldest daughter is pregnant, adding to the pressure. The family lives in a cramped two-bedroom house with damp problems affecting her youngest son’s asthma, but the landlord won’t help. Money is tight, and Gloria is considering a payday loan. A teacher has raised concerns about her middle son’s focus at school and dental hygiene. Though Gloria stays strong for her family, the stress is taking a toll, and she often feels overwhelmed and anxious. She has tried to book a GP appointment to seek for mental health support with no luck so far.”
*“Mo*,* a 32-year-old delivery driver*,* has been living in the UK for 18 months with his wife and three children. He enjoys spending time with friends at the local Mosque and is slowly improving his English*,* helped by his children. Mo is overweight and often relies on quick*,* unhealthy meals while working. Recently*,* he’s been experiencing back pain but keeps it to himself*,* worried about causing concern at home or appearing unfit to his employer. He avoids seeing a GP afraid a sick note could put his job at risk. He asked for some stronger painkillers at the local pharmacy but with no prescription he was given ibuprofen as the only alternative available for him.”*

### Data Analysis

Descriptive statistics were calculated to summarise demographic characteristics and HLQ scores for each of the nine domains. A hierarchical cluster analysis using Ward’s method with squared Euclidean distance was then conducted on HLQ responses. This technique is recommended in Ophelia-guided research to identify subgroups within the sample who share similar HL profiles [20, 22] Data was analysed using SPSS (v28).

Focus groups transcripts were analysed using thematic analysis, following the six-step approach by Braun and Clarke (2006) [23]. One researcher conducted the analysis, generating initial codes inductively and grouping them into themes through iterative review. Emerging themes were discussed with the wider research team to enhance rigour.

## Results

### Participant Characteristics

A total of 65 participants completed the Health Literacy Questionnaire (HLQ). The sample included individuals from African, Asian, Latin American, Afghan, and Ukrainian backgrounds. Most participants were female (64.6%), and nearly half were aged 18–39 years (47.7%). The largest ethnic group identified was Asian/Asian British (36.9%). The “Other ethnic group” category included participants identifying themselves as Mixed race (*n* = 3), Middle Eastern/ Middle Easter British- Arab, Turkish and Other (*n* = 10) (Table 2).

**Table 2:** Participant characteristics and HLQ scale scores

	*N* (%)
Gender	
Male	23 (35.4)
Female	42 (64.6)
Age	
18–39	31 (47.7)
40–59	26 (40.0)
60+	7 (10.8)
Prefer not to say	1 (1.5)
Ethnicity	
Asian/Asian British	24 (36.9)
Black/African/Caribbean/Black British	13 (20.0)
White- British, Irish, other	13 (20.0)
Other ethnic group	13 (20.0)
Prefer not to say	2 (3.1)
HLQ Scale	*Mean (SD)*
Feeling understood and supported by healthcare providers	2.52 (0.59)
Having sufficient information to manage my health	2.57 (0.55)
Actively managing my health	2.85 (0.57)
Social support for health	2.63 (0.53)
Appraisal of health information	2.70 (0.55)
Ability to actively engage with healthcare professionals	2.99 (0.87)
Navigating the healthcare system	2.91 (0.77)
Ability to find good health information	3.10 (0.80)
Understanding health information enough to know what to do	3.42 (0.77)

## HLQ Scale

**Fig. 1:**
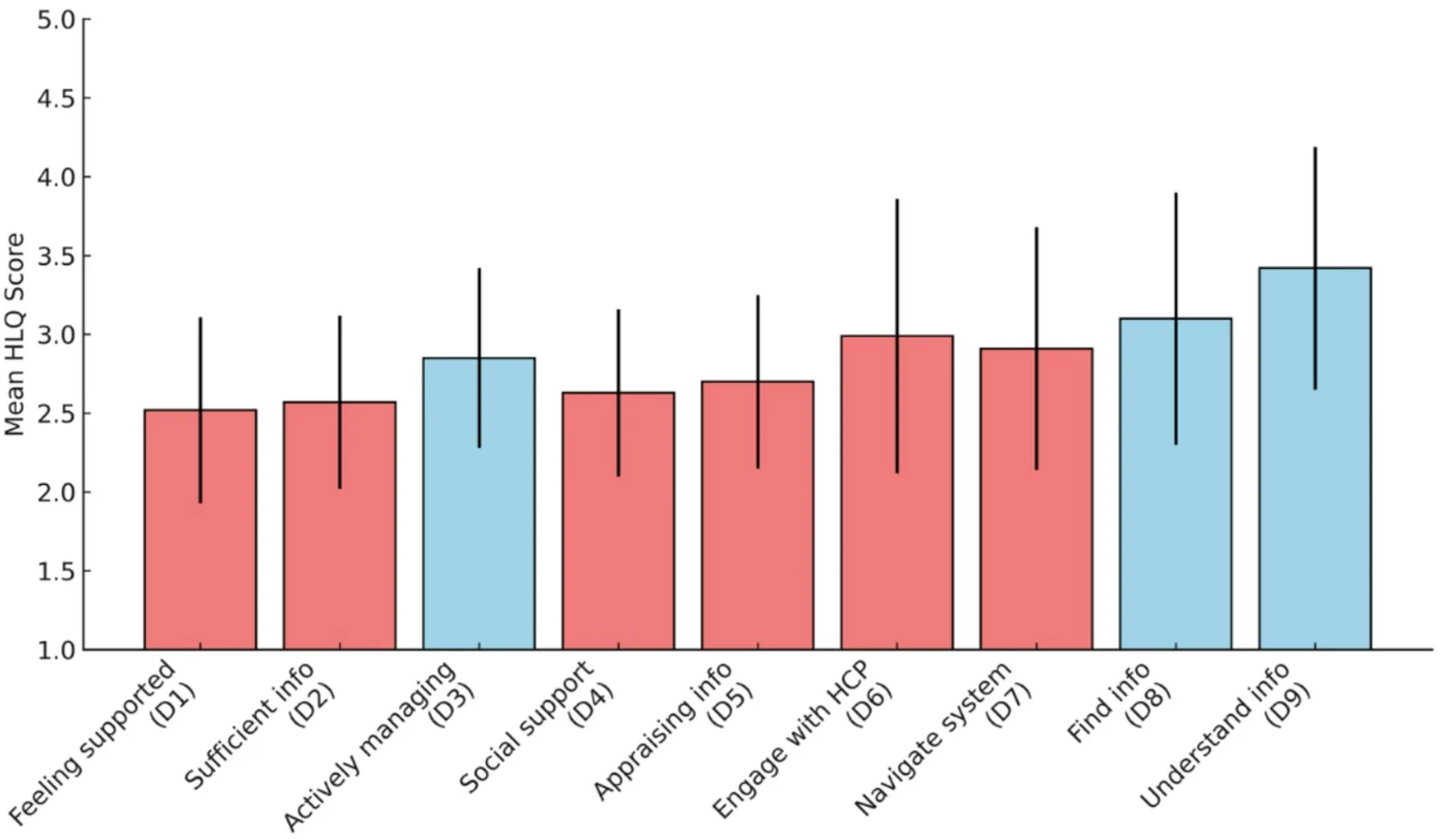
HLQ scale mean scores (SD) across nine domains. Blue bars indicate domains with relatively higher scores indicating greater ease or confidence within each domain; red bars indicate domains with lower scores where participants experienced greater difficulty

Participants reported moderate strengths in understanding and searching for health information (Domains 8 and 9). However, lower scores in feeling supported by healthcare providers, accessing sufficient information, social support for health, appraising the quality of health information, engaging with healthcare professionals and navigating the healthcare system (Domains 1, 2, 3, 4, 5, 6 and 7) indicate widespread issues with confidence, support, and communication when using health information, consistent with known barriers faced by migrant populations (Fig. 1). These findings suggests that although participants can locate and comprehend health content, they face challenges in judging its reliability and using it confidently within complex healthcare settings.

### Cluster Analysis

Cluster analysis identified four distinct health literacy profiles (Table 3). Cluster 1 (46%) demonstrated the strongest overall HL but felt somewhat less supported by healthcare providers. Cluster 2 (35%) showed moderate capabilities, particularly struggling with system navigation and confidence. Cluster 3 (12%) had low self-management and support yet scored highly in understanding and finding health information, suggesting good comprehension but limited capacity to act. Cluster 4 (7%) was the most vulnerable, with very low scores across all domains, indicating major barriers to accessing and using health information. Clusters 3 and 4 were selected for qualitative follow-up.

**Table 3:**
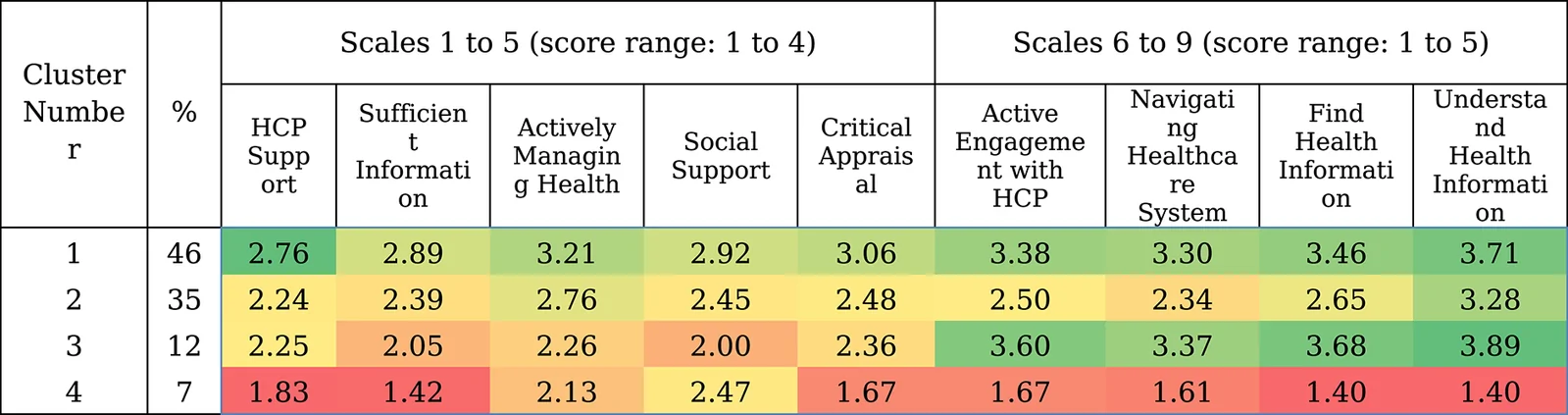
Health literacy profiles based on four-cluster solution(Colour-coded scores using a traffic-light system with green indicating relative strengths and red indicating areas of greater challenge. Patterns of HLQ domain scores represent the health literacy profile for each cluster)

### Focus Groups Findings

Two focus groups were conducted with participants purposively sampled from the two most vulnerable clusters (Clusters 3 and 4). A total of 14 individuals took part, including 6 male and 8 female in the 18–39 or 40–59 age brackets. Participants were assigned to groups based on shared cultural and linguistic backgrounds, resulting in one group with African heritage participants (*n* = 7) and one with Latin American participants (*n* = 7).

Participants exposed several themes regarding barriers to accessing and navigating healthcare services (Table 4). Both groups reported difficulties in booking appointments (especially via phone), confusion around NHS processes, limited support for those with low digital or English literacy, and a reliance on informal networks such as friends, family, or community groups. Emotional stress, often linked to past experiences or current circumstances (e.g. immigration status, isolation), intensify challenges in managing health. However, each group also expressed unique issues reflecting their specific cultural, linguistic, and structural experiences within the health system.

Latin American participants reported major barriers when trying to access healthcare, particularly when booking appointments by phone. *“Yes*,* I call my GP and I have to wait a long time. Sometimes I wait and they don’t even answer”* (*Participant L3*). Frustration was compounded by issues with dental access: *“I waited two years to get a dentist appointment. Then they cancelled my next one with no explanation”* (*Participant L6*). These challenges reflected limited ability to navigate the healthcare system. Language was a significant barrier, with participants often unable to understand NHS letters or manage online processes: *“She (my wife) says that she gets letters in English and she doesn’t understand”* (*Participant L1*). Even those with some English proficiency struggled to obtain interpreter support: *“They told me to come with a friend who can translate. I don’t have anyone*,* so they asked the cleaning lady to translate. I was really upset”* (*Participant L7*). These issues contributed to emotional distress and a sense of exclusion: *“It’s very hard when you don’t know what to do and you’re alone”* (*Participant L5*). To cope, many relied on family or informal support networks: *“My daughter did it for me (the appointment)*,* because it was online and I couldn’t do it”* (*Participant L2*). Although not captured in the transcript, the facilitator noted that some participants privately expressed concern that raising complaints or frustrations might affect their visa status. This fear may have limited their open engagement in the group discussion.

African participants, though generally fluent in English, also described communication challenges related to rushed consultations and perceived accent bias: *“Even if you speak English*,* sometimes they say they don’t understand you”* (*Participant A5*). This reflected low perceived support from healthcare providers and limited engagement. Interpreter services were often not offered: *“They should ask you if you want someone to help you. Not everyone will say it themselves because they don’t want to bother or loss the appointment”* (*Participant A1*). Participants also found digital and phone-based access pathways confusing and ineffective: *“They tell you to go online or to 111*,* but then nothing happens”* (*Participant A6*). Fragmented care and the lack of continuity undermined trust in the system: *“Every time*,* it’s someone new (a GP). They don’t know what happened before”* (*Participant A5*). As with the Latin American group, informal networks were crucial for navigating services: *“In Church*,* we help each other. If someone doesn’t know what to do*,* another person will guide them”* (*Participant A2*).

**Table 4:** Thematic analysis of health literacy barriers and coping strategies among Latino and African participants

	Subthemes	
	Latino participants	African participants
Theme 1: Structural barriers to accessing healthcare	Difficulty booking GP and dental appointments, often no answer or long hold times	Delays in GP access and confusion around telephone-based urgent care helplines
	Uncertainty about eligibility and procedures (e.g. dental or specialist care)	Confusion navigating NHS pathways and unclear referral processes
	Discouragement due to system inflexibility (e.g. no walk-ins, cancellations)	Frustration with inconsistent advice and unclear service pathways
Theme 2: Communication, language, and digital challenges	Limited English proficiency leads to difficulty understanding letters and forms	Accent-related misunderstandings despite English fluency
	Lack of interpreter support; patients expected to bring their own	Fast, rushed communication style from providers causes confusion
	Struggles with digital systems for booking and form-filling	Unclear digital instructions; difficulty using NHS online systems
Theme 3: Coping mechanisms and emotional impact	Emotional stress, anxiety, and fear of being judged or ignored	Emotional fatigue from having to retell health stories repeatedly
	Reliance on family, friends, and voluntary groups for support	Use of church and community networks for navigating care
	Hesitation to speak up due to low confidence or fear of dismissal	Lack of continuity in care limits trust and emotional safety

## Discussion

This study adds to the evidence on racial and ethnic health disparities by focusing on the lived experiences of African and Latin American migrants in England. While participants generally showed they could find and understand health information, their ability to engage with healthcare services was often limited by structural and relational barriers. These findings question the emphasis on individual-level health literacy in existing models [2] and instead point to the influence of wider systemic exclusion, mistrust in institutions, and insecure immigration status.

Participants’ difficulties with navigating services were not simply due to unfamiliarity but were shaped by racialised dynamics of access and power. For many, healthcare interactions were marked by language discordance, limited interpreter access, and emotionally unsafe environments. These conditions contributed to feelings of being dismissed, dehumanised, or unworthy of care; experiences that disproportionately affected asylum seekers and individuals with uncertain immigration status [24]. These narratives reflect a broader pattern of structural racism embedded in healthcare delivery and policy [25].

Our use of the Ophelia process enabled the identification context-specific barriers that standard assessments often overlook. Importantly, the integration of quantitative and qualitative methods revealed significant gaps in the emotional and relational dimensions of health literacy. For example, participants who scored moderately well on HLQ scales still described profound difficulties in interacting with providers or trusting health information. This mismatch underscores the need to redefine health literacy not as an individual attribute, but as a socially situated practice deeply influenced by power, culture, and institutional norms [17].

The findings carry important implications for public health practice and health system reform. Current interventions often assume that improving individual knowledge or digital skills is sufficient to reduce disparities. However, such approaches overlook how systemic neglect and cultural disconnects causes disengagement [26]. Health literacy initiatives for migrant communities must therefore be co-designed, equity-driven, and grounded in the realities of those most affected. Culturally safe interpreter services, trauma-informed care, community navigation programmes, and inclusive communication strategies are essential components of a more just healthcare system [25].

Finally, the emotional toll experienced by participants, particularly fear, frustration, and helplessness, highlights the need for healthcare environments that not only inform, but also empower. Addressing the social determinants of health literacy means confronting broader inequities in immigration policy, housing, language access, and digital inclusion.

As part of the next phases of the Ophelia process, we are progressing to co-design workshops and individual interviews with community members, followed by engagement with stakeholders such as GP practices and pharmacies to refine and implement health literacy interventions. These steps will inform the development and testing of interventions tailored to the needs identified in this study.

### Strengths and Limitations

A key strength of this study lies in its use of the Ophelia process, which enabled a culturally responsive, mixed-methods exploration of health literacy within migrant communities. By combining quantitative profiling through the HLQ with qualitative insights from focus groups conducted in participants’ native languages, the study captured both individual capacities and systemic barriers. The involvement of community organisations in recruitment and interpretation also enhanced trust and relevance, supporting more authentic engagement from underrepresented populations.

However, the study has some limitations. The sample size, while appropriate for exploratory analysis, was relatively small and geographically confined, which may limit generalisability beyond the participating communities in England. Local community organisations reported that recruitment required multiple points of engagement with their groups, reflecting the challenges of involving underserved populations; however, the exact number of individuals approached was not available. Additionally, the reliance on self-reported data introduces the potential for social desirability bias, especially in group settings where participants may have felt pressure to present certain views. While this study focused on Latin American and African participants, it is important to recognise that migrant communities are highly diverse, with differing languages, cultural norms, migration experiences, and health system familiarity. These differences can influence how HL challenges are experienced and addressed. Further research is needed to explore these variations across other underrepresented migrant populations.

## Conclusion

This study highlights the need to consider both individual abilities and systemic barriers when addressing health literacy among migrant and minority ethnic communities. While participants showed moderate skills in understanding and searching for health information, many faced challenges in accessing services, communicating with providers, and managing care confidently. Emotional factors, such as stress and feeling dismissed, further limited their ability to engage with the healthcare system.

The findings emphasise the importance of culturally appropriate, community-informed approaches. Improving interpreter access, simplifying care pathways, and offering both digital and non-digital options are key to reducing barriers. Involving communities in designing support tools and services will help ensure that interventions are relevant and effective.

Health literacy is not just about reading or understanding information; it is shaped by relationships, trust, and access. Efforts to improve equity in healthcare must recognise and respond to these broader social and structural factors.

## Supplementary Information

Below is the link to the electronic supplementary material.

Supplementary Material 1

## Data Availability

The datasets generated and analysed during the current study are available from the corresponding author on reasonable request.
