# Supplementary material for: Unpacking Health Literacy Disparities in Migrant and Ethnic Minority Communities in England
Source: J Immigr Minor Health. 2026 Feb 6;28(5):1106–13. doi: 10.1007/s10903-026-01861-8 (PMC13612664; doi:10.1007/s10903-026-01861-8)
Supplement: Supplementary file 1 [file 10903_2026_1861_MOESM1_ESM.pdf]

Supplementary File 1. Focus Group Vignettes and Questions Guide

CASE 1- Gloria

Gloria, a 40-year-old immigrant mother, takes pride in caring for her three children, whose ages range from 5 to 15. Her eldest, a 15-year-old daughter, is expecting Gloria's first grandchild, bringing both joy and additional challenges to their close-knit family. Despite Gloria's recent separation from her husband, she dedicates herself to working full-time in a warehouse to support her loved ones. The entire family shares a two-bedroom rental house, contributing to a sense of overcrowding.

Regrettably, Gloria has noticed damp patches around the house, causing concern for her youngest son's asthma. Struggling with timely rent payments, she is considering a payday loan. However, her landlord remains unsympathetic and neglectful regarding the house's maintenance and damp issues, adding to the family's challenges as newcomers with younger children.

Additionally, a teacher has recently raised concerns about Gloria's middle son's attention span during lessons and his poor dental hygiene.

Together with the teenage pregnancy of her eldest daughter adds another layer of complexity to the family's struggles. Gloria is now grappling with the potential challenges her pregnant daughter may face, from societal judgments to the need for additional support.

Despite her typically positive attitude, Gloria has found herself feeling teary and anxious at times, navigating the many challenges that arise in the context of being a young migrant family with younger children and a pregnant teenager.

CASE 1- Gloria (focus group questions as indicated by HLQ lower /higher scores)

Lower scores across the health literacy domains

Scale 1. Feeling understood and supported by healthcare providers

Lower scores: Your answer for this scale suggests that you've found it difficult to establish good relationships with healthcare providers. Has it always been this way? What would help you to improve these relationships?

Scale 2. Having sufficient information to manage my health

Lower scores: What would help you to feel confident that you have enough information about your health?

Scale 3. Actively managing my health

Lower scores: What do you need to help you manage your health differently?

Scale 4. Social support for health

Lower scores: What would help you to build relationships with family or friends?

Scale 5. Appraisal of health information

Lower scores: What would help you to understand health information and work out what is best for you?

Scale 6. Ability to actively engage with healthcare providers

Lower scores: How comfortable do you feel about talking with healthcare providers? What would help you to feel more comfortable to talk with them about your health?

Scale 7. Navigating the healthcare system

Lower scores: What would help you to find out about health support services that you need?

Scale 8. Ability to find good health information

Lower scores: What would help you to find information about your health?

Scale 9. Understanding health information well enough to know what to do

Lower scores: What would help you to understand written information about your health?

CASE 2 - Lucy and Ash

Lucy and Ash live on the same street.

Lucy is a 76-year-old lady who lives alone. She speaks limited English. Her husband died some years ago, and she has an adult daughter who lives nearby. Lucy doesn't like to ask her daughter for help to get to medical appointments as she knows she is busy with her own family. Lucy has Type 2 diabetes, arthritis and back pain, and most of the time feels quite low in her mood. She sees lots of different health professionals but struggles to find one she can really talk to and trust. Recently her doctor told her that her blood pressure was too high and that she needed to start a low-salt diet.

She was too afraid to ask him questions about this as he always seems in too much of a hurry, and now she is not sure where she can go for more help or information. She thinks maybe she should ask her elderly neighbour for advice. She is not sure that she can be bothered anyway to change her diet – it all seems too hard, and she has plenty of other things to worry about.

Ash is a 60-year-old man. Divorced for the past 10 years. Currently, all his children live and work away from the area.
In an effort to maintain good health, Ash walks three times a week, aspiring to do more despite finding it challenging to fit into his schedule. He consults with a male general practitioner but sometimes senses a hint of impatience when discussing his heartburn symptoms with the GP. To better manage his digestive symptoms, Ash explores various information sources, occasionally turning to the internet for more insights. However, he grapples with discerning what is relevant to his situation and whether the information he comes across is trustworthy.

CASE 2 - Lucy and Ash (focus group questions as indicated by HLQ lower /higher scores)
Fairly confident in their ability to use the healthcare system and seek advice when they need it. However, they don't feel supported by healthcare providers, don't feel they have enough information about their health (domain 2) and are confused about mixed messages. They aren't proactive about managing their health and feel they don't have sufficient social support to help them think this through.

Scale 1. Feeling understood and supported by healthcare providers
Lower scores: Your answer for this scale suggests that you've found it difficult to establish good relationships with healthcare providers. Has it always been this way? What would help you to improve these relationships?
Scale 2. Having sufficient information to manage my health
Lower scores: What would help you to feel confident that you have enough information about your health?
Scale 3. Actively managing my health
Higher scores: How did you learn to manage your health? What do you do to manage your health?
Scale 4. Social support for health
Lower scores: What would help you to build relationships with family or friends?
Scale 5. Appraisal of health information
Lower scores: What would help you to understand health information and work out what is best for you?
Scale 6. Ability to actively engage with healthcare providers
Higher scores: How comfortable do you feel about talking about your health with healthcare providers? What has helped you with this?
Scale 7. Navigating the healthcare system
Lower scores: What would help you to find out about health support services that you need?
Scale 8. Ability to find good health information
Higher scores: What has helped you to find information about your health?
Scale 9. Understanding health information well enough to know what to do
Lower scores: What has helped you to understand written health information?

CASE 3 - Rosalyn & Mike

Rosalyn and Mike are a couple.
Rosalyn is 24 years old and is married with 2 children. She is very sociable and enjoys spending time with friends and family. She works as a volunteer at the local food bank.
She had a great relationship with both her midwife and health visitor when she first had her children although as they are now aged 6 and 7 she no longer sees the health visitor.
Rosalyn has always managed her health well but she recently found a lump in her breast which concerns her. She has always felt well supported by her GP but isn't sure what to do next.
Mike is 26 years old and works in IT as a Computer Repairer. He likes to keep fit and regularly goes to the gym although he has smoked since he was at school. He lives with his wife and two children. His youngest son has recently been diagnosed with asthma.
Mike never goes to the GP and leaves all the health care issues to his wife as he is working. He is considering giving up smoking now as he realises it's not good for his son if he continues to smoke. However, many of his colleagues at work smoke and so he thinks this will make it very difficult to give up.

CASE 3- Rosalyn & Mike (focus group questions as indicated by HLQ lower /higher scores)
Able to manage their health pretty well and feel they have good social support and information and feel supported by healthcare providers. However, they aren't sure where to go to find health information, understand it or how to navigate the healthcare system.

Scale 1. Feeling understood and supported by healthcare providers
Higher scores: Your answers for this scale suggest you have good relationships with your healthcare providers – is this how you see it? Why is this?
Scale 2. Having sufficient information to manage my health
Higher scores: What helps you to feel you have enough information to manage your health and make

decisions?

Scale 3. Actively managing my health

Higher scores: How did you learn to manage your health? What do you do to manage your health?

Scale 4. Social support for health

Higher scores: What has helped you to build a strong system of support from family and friends?

Scale 5. Appraisal of health information

Lower scores: What would help you to understand health information and work out what is best for you?

Scale 6. Ability to actively engage with healthcare providers

Higher scores: How comfortable do you feel about talking about your health with healthcare providers? What has helped you with this?

Scale 7. Navigating the healthcare system

Lower scores: What would help you to find out about health support services that you need?

Scale 8. Ability to find good health information

Higher scores: What has helped you to find information about your health?

Scale 9. Understanding health information well enough to know what to do

Lower scores: What has helped you to understand written health information?

CASE 4 - Mo

Mo is 32 years old and works part-time as a delivery driver. He has been in the UK for 18 months and lives with his wife and three children. He likes to spend time with friends at the local Mosque.

He now speaks a little English and is gradually picking up more as his children speak some English at home. Mo is overweight, but as he is always on the road, his only options for lunch are sandwiches and snacks. He has recently started to struggle with back pain although doesn't want to share this with anyone as he knows his family will worry and his boss may think he is unfit to work.

He also doesn't want to see his GP about this as he sees someone different each time he goes and thinks they will only want to give him a sick note which might mean he loses his job.

CASE 4- Mo (focus group questions as indicated by HLQ lower /higher scores)

Fairly confident in their ability to use the healthcare system and seek advice when they need it. However, they don't feel supported by healthcare providers, don't feel they have enough information about their health and are confused about mixed messages. They aren't proactive about managing their health and feel they don't have sufficient social support to help them think this through

Scale 1. Feeling understood and supported by healthcare providers

Higher scores: Your answers for this scale suggest you have good relationships with your healthcare providers – is this how you see it? Why is this?

Scale 2. Having sufficient information to manage my health

Higher scores: What helps you to feel you have enough information to manage your health and make decisions?

Scale 3. Actively managing my health

Higher scores: How did you learn to manage your health? What do you do to manage your health?

Scale 4. Social support for health

Higher scores: What has helped you to build a strong system of support from family and friends?

Scale 5. Appraisal of health information

Lower scores: What would help you to understand health information and work out what is best for you?

Scale 6. Ability to actively engage with healthcare providers

Higher scores: How comfortable do you feel about talking about your health with healthcare providers? What has helped you with this?

Scale 7. Navigating the healthcare system

Lower scores: What would help you to find out about health support services that you need?

Scale 8. Ability to find good health information

Higher scores: What has helped you to find information about your health?

Scale 9. Understanding health information well enough to know what to do

Lower scores: What has helped you to understand written health information?

CASE 5 - Mary

Mary is 45 years old, married and has 4 children. She was a teenager when she got married and she fell pregnant soon after so left school early to look after her family.

Her oldest two children have left home now and Mary has one grandchild who she regularly looks after. Mary is

a heavy smoker and is overweight which concerns her as she wants to be fit and healthy to play with her grandchild.

When she visited her GP Practice recently for her contraceptive check the Practice Nurse commented on her smoking and asked if she would consider quitting. Maria would like to try to stop but is worried that she may gain more weight as a result.

CASE 5 – Mary (focus group questions as indicated by HLQ lower /higher scores)

Fairly average scores across the health literacy domains. They feel they have a good understanding of healthcare systems and health information and engagement with providers

Scale 1. Feeling understood and supported by healthcare providers

Lower scores: Your answer for this scale suggests that you've found it difficult to establish good relationships with healthcare providers. Has it always been this way? What would help you to improve these relationships?

Scale 2. Having sufficient information to manage my health

Lower scores: What would help you to feel confident that you have enough information about your health?

Scale 3. Actively managing my health

How did you learn to manage your health? What do you do to manage your health?

Scale 4. Social support for health

What has helped you to build a strong system of support from family and friends?

Scale 5. Appraisal of health information

Lower scores: What would help you to understand health information and work out what is best for you?

Scale 6. Ability to actively engage with healthcare providers

Lower scores: How comfortable do you feel about talking with healthcare providers? What would help you to feel more comfortable to talk with them about your health?

Scale 7. Navigating the healthcare system

Lower scores: What would help you to find out about health support services that you need?

Scale 8. Ability to find good health information

Lower scores: What would help you to find information about your health?

Scale 9. Understanding health information well enough to know what to do

Lower scores: What would help you to understand written
